# *Myxococcus xanthus* predation: an updated overview

**DOI:** 10.3389/fmicb.2024.1339696

**Published:** 2024-01-24

**Authors:** Francisco Javier Contreras-Moreno, Juana Pérez, José Muñoz-Dorado, Aurelio Moraleda-Muñoz, Francisco Javier Marcos-Torres

**Affiliations:** Departamento de Microbiología, Facultad de Ciencias, Universidad de Granada, Granada, Spain

**Keywords:** myxobacteria, bacterial predation, bacterial interaction, predator-prey interactions, motility, secondary metabolites, hydrolytic enzymes, metals

## Abstract

Bacterial predators are widely distributed across a variety of natural environments. Understanding predatory interactions is of great importance since they play a defining role in shaping microbial communities in habitats such as soils. *Myxococcus xanthus* is a soil-dwelling bacterial predator that can prey on Gram-positive and Gram-negative bacteria and even on eukaryotic microorganisms. This model organism has been studied for many decades for its unusual lifecycle, characterized by the formation of multicellular fruiting bodies filled with myxospores. However, less is known about its predatory behavior despite being an integral part of its lifecycle. Predation in *M. xanthus* is a multifactorial process that involves several mechanisms working synergistically, including motility systems to efficiently track and hunt prey, and a combination of short-range and contact-dependent mechanisms to achieve prey death and feed on them. In the short-range attack, *M. xanthus* is best known for the collective production of secondary metabolites and hydrolytic enzymes to kill prey and degrade cellular components. On the other hand, contact-dependent killing is a cell-to-cell process that relies on Tad-like and type III secretion systems. Furthermore, recent research has revealed that metals also play an important role during predation, either by inducing oxidative stress in the prey, or by competing for essential metals. In this paper, we review the current knowledge about *M. xanthus* predation, focusing on the different mechanisms used to hunt, kill, and feed on its prey, considering the most recent discoveries and the transcriptomic data available.

## Introduction

*Myxococcus xanthus* is a soil-dwelling bacterial predator renowned for its social and multicellular behavior, which is evident along its complex lifecycle. When nutrients are scarce, *M. xanthus* cells coordinate to form multicellular structures known as fruiting bodies, where some of them differentiate into resistant myxospores. Depending on external cues, such as nutrient levels or the presence of prey microorganisms, *M. xanthus* must decide whether to initiate this developmental cycle or to activate its predatory mechanisms to feed (Muñoz-Dorado et al., 2016).

Coordinating these two facets of its lifecycle has led to an extensive coevolution between both stages. Indeed, mutations in genes involved in early stages of the developmental cycle, where cells are still to commit to this process, have been found to negatively impact predation. By contrast, genes required in later stages of the development, where cells are fully committed to fruiting-body formation, do not seem to play a role in predation (Pham et al., 2005; Berleman et al., 2008; Pérez et al., 2022). Furthermore, some predator–prey interactions can stimulate fruiting bodies formation even during predation, although cells within the fruiting bodies are unable to differentiate into myxospores (Berleman and Kirby, 2007).

**Figure 1:**
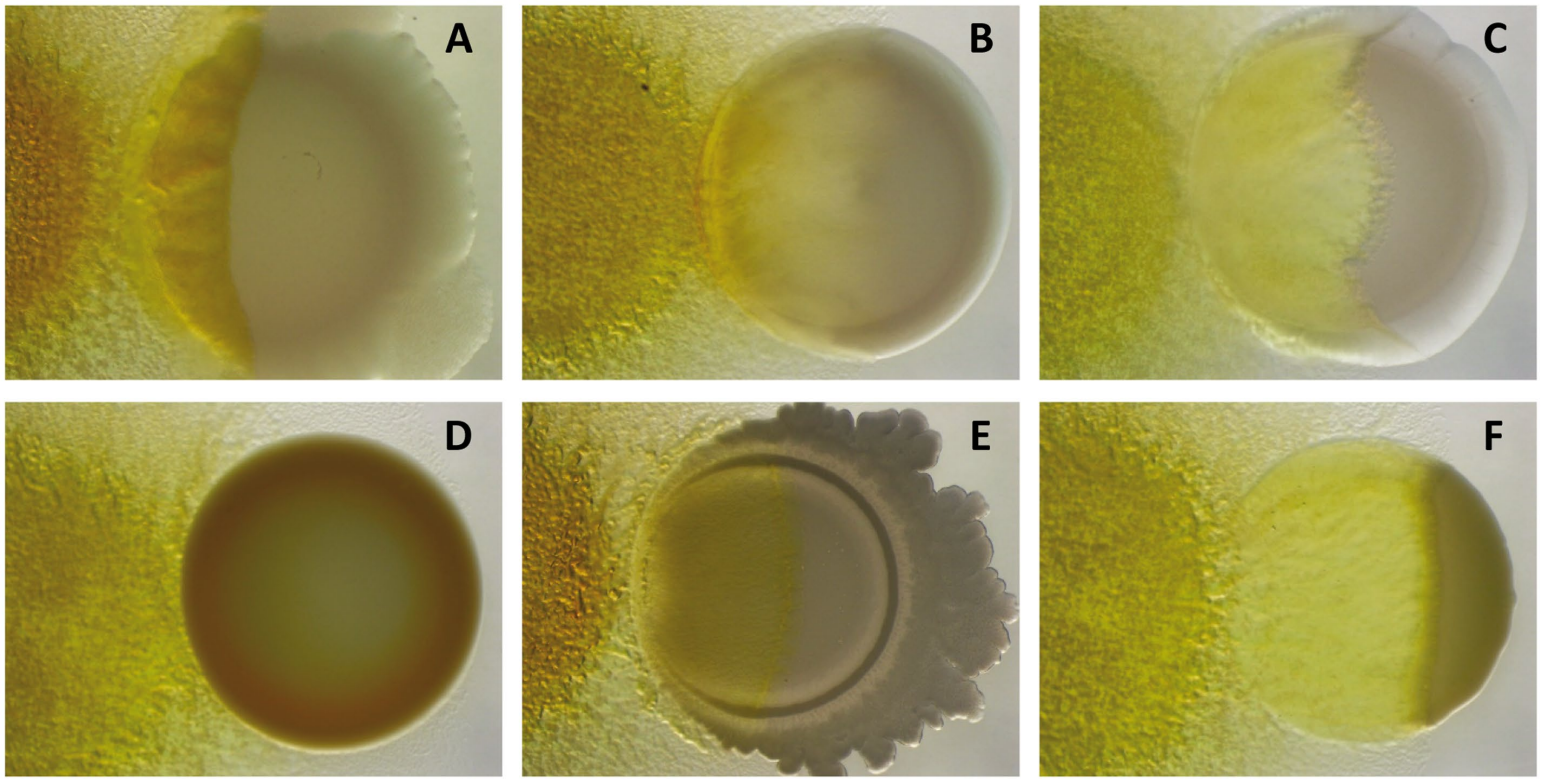
Predation assays of *M. xanthus* DK1622 (left) against different Gram-negative [**(A)** *Pseudomonas putida*; **(B)** *Sinorhizobium meliloti*; **(C)** *Escherichia coli*] and Gram-positive bacteria [**(D)** *Staphylococcus aureus*; **(E)** *Bacillus subtilis*; **(F)** *Micrococcus* sp.] after 72 h of interaction, showing its different capabilities to kill and consume prey.

When conditions are favorable again, myxospores from a fruiting body will germinate into a population of vegetative cells known as swarm, which will actively hunt for prey to feed on them. Cells within the swarm will cooperate in an attempt to prey with different degrees of success on a great diversity of Gram-negative and Gram-positive bacteria (Figure 1), including nitrogen-fixing bacteria and some human and plant pathogens, as well as fungi and nematodes (Mendes-Soares and Velicer, 2013; Livingstone et al., 2017; Petters et al., 2021; Sydney et al., 2021).

*M. xanthus* predation is a multifactorial task that combines a broad arsenal of resources to ensure prey death. This process starts with tracking of prey in the environment driven by its motility systems and signal-transduction mechanisms. Upon encountering a suitable prey, *M. xanthus* uses a combination of short-range and contact-dependent mechanisms to kill and lyse prey cells. While short-range killing mainly involves the production of a battery of secondary metabolites (SMs) such as antibiotics, and of hydrolytic enzymes to degrade and feed on the cellular components of the prey (Muñoz-Dorado et al., 2016), contact-dependent lysis is mediated by secretion systems (Seef et al., 2021; Thiery et al., 2022). Moreover, recent studies have shown that metals are also involved in *M. xanthus* predation either by using metals to provoke oxidative stress or by outcompeting prey for possession of essential metals (Contreras-Moreno et al., 2020; Lee et al., 2020; Dong et al., 2022b; Pérez et al., 2022).

While the developmental stage of *M. xanthus* has been thoroughly studied, less attention has been paid to its predatory behavior. This is, however, not an isolated facet of its lifestyle, but is heavily interconnected to the other traits of *M. xanthus* biology to shape its lifecycle (Volz et al., 2012; Pérez et al., 2022). In fact, there is mounting evidence that predation has played a major role in the selection of *M. xanthus* biological features via coevolution with its prey (Nair et al., 2019; La Fortezza et al., 2022). In this minireview we will discuss the state-of-the-art of the toolset used by *M. xanthus* to prey, including the most recent findings derived from transcriptomic analyses during predation.

## Motility systems

*M. xanthus* cells must actively search for prey in the soil to obtain nutrients. To approach the prey, they use two types of motility systems: an individual gliding movement, known as adventurous (A) motility, and a collective twitching-type movement, known as social (S) motility. A-motility relies on a Agl-Glt multiprotein outer-membrane complex that attaches the substrate at fixed sites of focal adhesion. These Agl-Glt complexes move directionally across the inner membrane toward the anterior pole of the cell, following a helical trajectory (Islam et al., 2023). Gliding occurs over an exopolysaccharide slime produced by the bacterium, which facilitates cells to follow the trail of previous cells rather than creating a new one, which enables exploration and prey foraging (Rombouts et al., 2023). On the other hand, S-motility is driven by type-IV pili, which pull the cells forward by extending, attaching to surfaces (or other cells), and then retracting (Chang et al., 2016). In *M. xanthus*, this is a collective movement where the cells must be in contact with each other, allowing them to coordinate the swarm’s movement (Skotnicka and Søgaard-Andersen, 2017).

A study using mutants impaired in these two motility systems clearly showed that both are required to efficiently prey on *Sinorhizobium meliloti* (Pérez et al., 2014). In fact, transcriptomic analyses of *M. xanthus* during predation (predatosomes) have shown upregulation of some genes required for both motility systems as well as some regulators involved in motility, such as the sigma factor SigF (Lee et al., 2020; Pérez et al., 2022). Both types of motility occur simultaneously or alternate in different subpopulations of the swarm to adapt to different prey local distribution (Rombouts et al., 2023).

While scouting the area in search for prey, *M. xanthus* seems to detect some prey molecules such as acyl homoserine lactones, that stimulate motility and facilitate their encounter (Lloyd and Whitworth, 2017; Akbar et al., 2022). Once the predator finds its prey, it must stay in close vicinity to activate its attack mechanisms and then feed on their cellular by-products. Therefore, upon detection, *M. xanthus* cells “stop” by repeatedly reversing their trajectory to optimize prey lysis (Zhang et al., 2020; Thiery and Kaimer, 2022). These repeated reversals are also important to feed on prey, as mutant predator cells defective in this mechanism tend to abandon prey colonies after lysis, without consuming their cellular remains (McBride and Zusman, 1996; Zhang et al., 2020).

## Secondary metabolites

**Figure 2:**
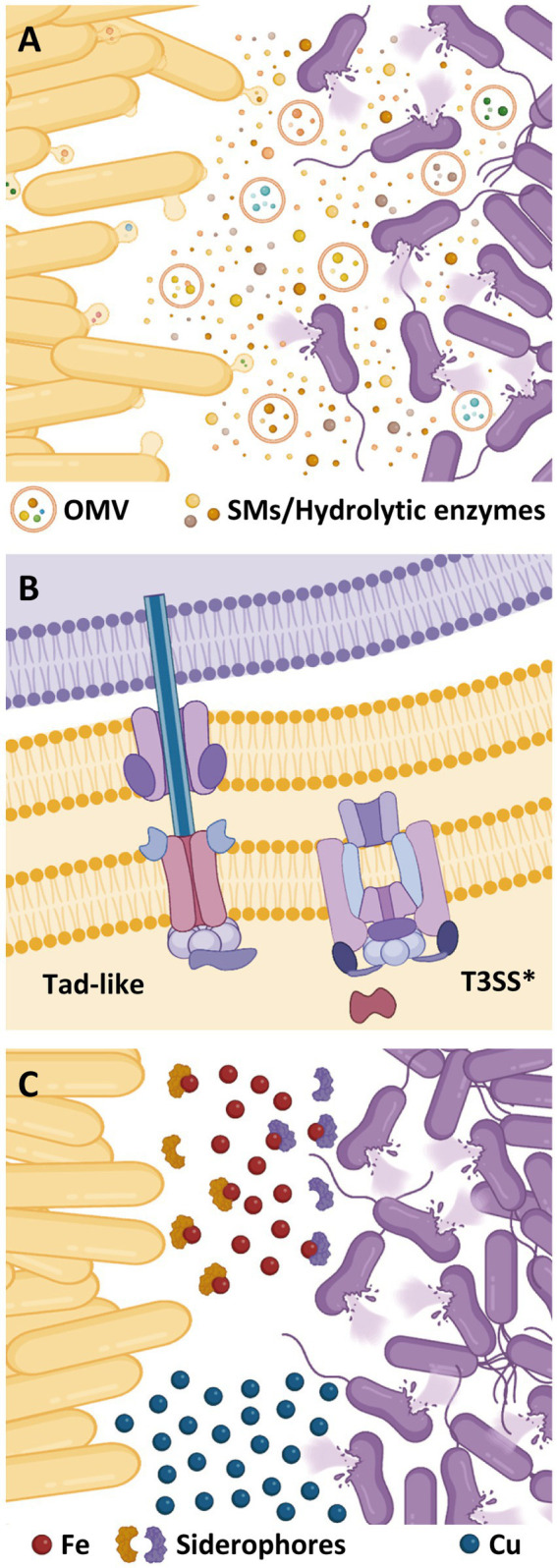
Different resources used by *M. xanthus* (yellow cells) to kill prey (purple cells). **(A)** Secondary metabolites (SMs) and hydrolytic enzymes are secreted into the extracellular medium or delivered via outer membrane vesicles (OMVs). **(B)** Contact-dependent killing mechanisms of *M. xanthus*. **(C)** Role of metals during bacterial predation. Competition for iron mediated by siderophores and accumulation of copper at the predator–prey interphase.

*M. xanthus* induces prey cell death and lysis by cooperative production of different lytic factors, acting either in isolation or synergistically (Figure 2A). Among them, SMs play an important role in prey killing, especially those with antimicrobial activity. *M. xanthus* genome holds an outstanding biosynthetic capacity for SM production, including at least 18 nonribosomal peptide synthetases (NRPS), 22 polyketide synthases (PKS), and 6 mixed PKS/NRPS, making a total of 14.5% of its genome (Goldman et al., 2006). Antimicrobial compounds so far isolated from *M. xanthus* have shown to be more efficient against Gram-positive bacteria (Xiao et al., 2011; Hoffmann et al., 2018), which could be due to the protective role of the Gram-negative outer membrane and/or a potential facilitation for the intracellular delivery of outer membrane vesicles (OMVs) cargo molecules (Zwarycz et al., 2023). To date, only 2 *M. xanthus* SMs have been directly implicated in predation: (i) myxovirescin, a macrocyclic SM able to block bacterial growth by inhibiting type a II signal peptidase (Xiao et al., 2011, 2012), and (ii) myxoprincomide, a SM required for effective predation against *Bacillus subtilis* (Cortina et al., 2012; Müller et al., 2016). However, the most recent predatosome data suggest that SM production is prey specific. Thus, while genes coding for myxoprincomide, myxovirescin, and myxalamide have been reported as being upregulated against *Micrococcus luteus* and *Escherichia coli*, only myxalamide is upregulated when preying on *S. meliloti*. However, against *S. meliloti*, additional clusters probably involved in the biosynthesis of unidentified bio-products are also upregulated (Pérez et al., 2022; Wang et al., 2023).

## Hydrolytic enzymes

Besides SMs, *M. xanthus* requires a battery of hydrolytic enzymes to degrade prey cell components and feed on them (Figure 2A). To induce lysis of Gram-positive bacteria, this myxobacterium needs to degrade prey cell-walls via peptidoglycan-lysing enzymes (Hart and Zahler, 1966; Sudo and Dworkin, 1972; Arend et al., 2021). A protein with lysozyme-like-activity (LlpM) able to induce cell lysis has been identified, although it is not essential for this process, reinforcing the idea that cell-wall lytic activity of the *M. xanthus* secretome is a multifactorial process with several hydrolytic enzymes involved (Arend et al., 2021). In fact, secretion of hydrolytic enzymes has shown to drive predation in natural isolates with genomes specially enriched in this type of enzymes (Dong et al., 2022a, 2023).

Transcriptomic studies against different prey have revealed upregulation of several *M. xanthus* genes coding cell-wall lysing proteins. Thus, when the Gram-negative *S. meliloti* and the Gram-positive *Streptomyces coelicolor* were used as prey, two genes coding N-acetylmuramoyl-L-alanine amidases were upregulated (Lee et al., 2020; Pérez et al., 2022). Moreover, five genes coding for proteins with LysM-domains were overexpressed against *S. meliloti* and two of them were also upregulated in co-cultures with *E. coli* (Livingstone et al., 2018; Pérez et al., 2022). However, during predation on *Pseudomonas aeruginosa* or *M. luteus*, no gene involved in cell-wall lysis was differentially expressed (Wang et al., 2023). These discrepancies may be due to characteristics of the prey, but also to variances in the methodology used on each assay.

In addition to cell-walls, *M. xanthus* must also hydrolyze prey proteins and lipids to use them as nutrients or as building blocks for metabolic processes. Until now, only the peptidase MepA has been experimentally studied and it seems to contribute to predation by degrading proteins released from lysed cells (Berleman et al., 2014). Indeed, *mepA* is upregulated in co-culture with *S. meliloti* and with *S. coelicolor*, supporting its role in predation (Lee et al., 2020; Pérez et al., 2022). *M. xanthus* predatosomes against different bacteria indicate that, to achieve full prey lysis, the predator needs to induce a plethora of hydrolytic enzymes. In fact, besides those aforementioned, *M. xanthus* genes coding for several hydrolytic enzymes are transcriptionally upregulated in co-culture with *S. meliloti*, *E. coli*, or *M. luteus*, including peptidases, metalloproteases, alpha/beta fold hydrolases, lipases, and nucleases (Pérez et al., 2022; Wang et al., 2023).

The delivery of this lethal cocktail to the prey is facilitated by OMVs, which include in their cargo many putative hydrolytic proteins and molecules associated with antibiotic activities (Kahnt et al., 2010; Berleman et al., 2014; Remis et al., 2014). In fact, isolated *M. xanthus* OMVs can kill Gram-positive and Gram-negative bacteria (Evans et al., 2012; Remis et al., 2014; Livingstone et al., 2018; Zwarycz et al., 2023). Moreover, it has been proved that OMV killing activity against different bacteria correlates with the predatory activity of *M. xanthus*. However, the absence of correlation between OMV killing activity and their ability to fuse with different prey cell-walls reinforces the idea that the composition of the OMVs cargo is more critical than their delivery for the predatory activity (Zwarycz et al., 2023).

## Contact-dependent killing

Although the predatory strategy of *M. xanthus* is usually described as a cooperative process, single myxobacteria can kill individual prey cells (Zhang et al., 2020; Arend et al., 2021; Seef et al., 2021).

This one-to-one interaction requires a contact-dependent mechanism (Figure 2B). In this sense, many bacteria have evolved specialized nanomachines to export proteins and/or virulence factors across the cell envelope into the surroundings, or to inject them into eukaryotic or prokaryotic cells (Costa et al., 2015; Galán and Waksman, 2018).

Although analyses of the *M. xanthus* genome have revealed a large potential for secretion (Konovalova et al., 2010), only two secretion systems have been so far related to cell contact-dependent prey killing: a degenerate type III-like system (T3SS*) and a tight adherence (Tad) secretion apparatus, also known as “Kil complex” (Seef et al., 2021; Thiery et al., 2022).

T3SSs are multiprotein complexes encoded by a broad range of bacteria with pathogenic or interbacterial antagonism (Galán and Waksman, 2018). While several pathogenic enterobacteria use specialized T3SS to deliver effector proteins into eukaryotic cells (Wagner et al., 2018), other species have adapted flagella-type T3SS for cytotoxin export (Dongre et al., 2018; Halte and Erhardt, 2021). However, *M. xanthus* T3SS* lacks a dedicated outer membrane secretin and any homologs to needle and translocon components (Figure 2B), thus being classified as “non-flagellar, needle-less” T3SS (Konovalova et al., 2010; Abby and Rocha, 2012; Diepold and Armitage, 2015).

On the other hand, Tad-like secretion systems are considered members of the type IV filament superfamily, and are related to bacterial adhesion, biofilm stabilization, and contact-dependent regulation of adhesion (Ellison et al., 2017; Denise et al., 2019; Seef et al., 2021).

*M. xanthus* T3SS* and Tad-like components have been shown to be interdependently, but coordinately, accumulated at the predator–prey interface for killing *E. coli* prey cells, exhibiting a functional interplay and different functions during the predatory interaction. Thus, while the Tad-like apparatus is instrumental in prey cell death (but does not cause prey disintegration), the T3SS* is required for cell lysis (Thiery et al., 2022). Nevertheless, both systems are required for utilizing live prey as a nutrient source, although they are not directly involved in the degradation or uptake of dead prey biomass (Thiery et al., 2022).

Homologous genes encoding Tad-like complexes have been identified in different genera of bacterial predators (Seef et al., 2021; Wang et al., 2023). However, the co-occurrence of T3SS* and Tad complexes is restricted to the sub-order *Cystobacterineae* of the *Myxococcales*, suggesting a specialized function of these secretion systems in myxobacterial predation (Abby and Rocha, 2012; Wang et al., 2023).

Gram-negative and Gram-positive prey elicit similar responses by *M. xanthus* regarding the formation of Tad and T3SS* foci, since both can lead to aggregation of these multiprotein complexes (Wang et al., 2023). Thus, the transcriptomic analysis of the co-culture of *M. xanthus* with *E. coli* and *M. luteus* revealed that part of the genes encoding the T3SS*, as well as part of one of the two clusters encoding the Tad-like apparatus, were significantly upregulated in both cases (Wang et al., 2023). A similar result was reported from the interaction between *M. xanthus* and *S. meliloti*, although in this case the upregulation of genes from both clusters coding the Tad-like complex was detected (Pérez et al., 2022). However, during co-culture with *S. coelicolor*, transcriptomic data indicated that only some genes coding the T3SS*, but not those coding the Tad-like apparatus, were upregulated (Lee et al., 2020). Similarly, *P. aeruginosa* failed to induce accumulation of Tad complexes, turning out to be resistant to *M. xanthus* predation (Seef et al., 2021). Interestingly, the coordinated accumulation of the T3SS*/Tad complexes has not been observed during contact with other *M. xanthus* cells, but only during the interaction with prey cells, which implies that contact-dependent killing mechanisms discriminate between kin and prey cells (Wall, 2016; Seef et al., 2021; Thiery et al., 2022).

## Role of metals in the predatory interaction

The use of metals by bacterial predators has gained relevance in the recent years as a new mechanism of inducing prey death. Two metals have been reported to be involved in the predatory behavior of *M. xanthus*: copper and iron (Figure 2C). These two metals exhibit a dual role on living cells since they are cofactors of enzymes that are essential for vital functions, but toxic at high concentrations (Rensing and McDevitt, 2013; Ladomersky and Petris, 2015; Li et al., 2021).

In the case of copper, it has been shown that *M. xanthus* utilizes this metal to poison *S. meliloti*. This metal accumulates inside the cells at the interface where predator collides with the prey, provoking changes in both partners of the interaction and helping the predator to penetrate in the prey colony (Contreras-Moreno et al., 2020). While *M. xanthus* upregulates the expression of copper detoxification genes such as those coding for the P1B-ATPase CopA, the multicopper oxidase CuoA, and the copper efflux pumps Cus2 and Cus3 (Sánchez-Sutil et al., 2007; Moraleda-Muñoz et al., 2010a,b; Pérez et al., 2018), *S. meliloti* produces a brown pigment that has been identified as melanin (Contreras-Moreno et al., 2020). Predatory analyses have revealed that melanin is overproduced by the prey during predation, indicating that copper is being used to generate oxidative stress and that the pigment functions as a defensive shield for the prey (Contreras-Moreno et al., 2020). However, it remains to be elucidated how the predator achieves accumulation of copper in the prey to kill it by generating reactive oxygen species.

Iron also seems to play an important role during myxobacterial predation. Several transcriptomes of *M. xanthus* against diverse prey have been published, and in all of them siderophore biosynthesis is upregulated in both predator and prey (Lee et al., 2020; Pérez et al., 2022; Soto et al., 2023; Wang et al., 2023; Whitworth et al., 2023). Moreover, the same result has been observed during predation of another myxobacterium, *Cystobacter ferrugineus*, against *Pseudomonas putida* (Akbar and Stevens, 2021), indicating that competition for iron may be a general predatory mechanism among myxobacteria.

Experimentally, it has been demonstrated that depletion of iron triggers the biosynthesis of the antibiotic actinorhodin in *S. coelicolor* to prevent predation from *M. xanthus* (Lee et al., 2020). Moreover, a mutant of *M. xanthus* that produces less siderophores (myxochelins) is defective in predation against *P. aeruginosa* (Dong et al., 2022b). Similarly, mutants in a putative TonB-dependent transporter for ferrimyxochelins and in components of the ABC transporter that introduces ferrimyxochelins into the cytoplasm also exhibit less efficient predation on this prey (Dong et al., 2022b). All these data seem to indicate that competition for iron plays a decisive role in the myxobacterial predatory interaction with the prey.

## Concluding remarks

Bacterial predation is a key factor in shaping ecosystems and establishing microbial diversity in soils. Understanding these interactions will contribute to improve soil conditions in agriculture. Moreover, some bacterial predators are considered “microfactories” of SMs that can be used to overcome the current antibiotic crisis (Pérez et al., 2016, 2020).

Predation is an integral part of *M. xanthus* biology and, therefore, this bacterium has developed a diverse toolset to adapt to its predatory lifestyle. Among the best-understood facets of its predatory activity are the mechanisms used to kill and lyse their prey. The cooperative production of SMs and hydrolytic enzymes along with cell-to-cell contact killing via T3SS* and Tad-like complexes, are well-established predatory mechanisms. Future research in this topic will provide new information about less known aspects of *M. xanthus* predation and uncover new tools used by this microorganism to kill prey. In fact, recent studies have led to the identification of metals as additional weapons used by myxobacteria to kill their prey. Since fluctuations in metal concentration may occur in the habitats because of several activities, it is expected that they determine which population will predominate in the myxobacterial predatory interaction, which may have a significant impact on the environment and agriculture.

## Author contributions

FC-M: Writing – original draft, Writing – review & editing. JP: Writing – original draft, Writing – review & editing. JM-D: Writing – original draft, Writing – review & editing, Funding acquisition. AM-M: Writing – original draft, Writing – review & editing, Funding acquisition. FM-T: Conceptualization, Writing – original draft, Writing – review & editing, Funding acquisition.

## References

[ref1] Abby S. S. Rocha E. P. C. (2012). The non-flagellar type III secretion system evolved from the bacterial flagellum and diversified into host-cell adapted systems. PLoS Genet. 8:e1002983. doi: 10.1371/journal.pgen.1002983, PMID: 23028376 PMC3459982

[ref2] Akbar S. Phillips K. E. Misra S. K. Sharp J. S. Stevens D. C. (2022). Differential response to prey quorum signals indicates predatory specialization of myxobacteria and the ability to predate *Pseudomonas aeruginosa*. Environ. Microbiol. 24, 1263–1278. doi: 10.1111/1462-2920.15812, PMID: 34674390 PMC9257966

[ref3] Akbar S. Stevens D. C. (2021). Functional genomics study of *Pseudomonas putida* to determine traits associated with avoidance of a myxobacterial predator. Sci. Rep. 11:16445. doi: 10.1038/s41598-021-96046-8, PMID: 34385565 PMC8360965

[ref4] Arend K. I. Schmidt J. J. Bentler T. Lüchtefeld C. Eggerichs D. Hexamer H. M.. (2021). *Myxococcus xanthus* predation of gram-positive or gram-negative bacteria is mediated by different bacteriolytic mechanisms. Appl. Environ. Microbiol. 87, e02382–e02320. doi: 10.1128/aem.02382-20 33310723 PMC8090889

[ref5] Berleman J. E. Allen S. Danielewicz M. A. Remis J. P. Gorur A. Cunha J.. (2014). The lethal cargo of *Myxococcus xanthus* outer membrane vesicles. Front. Microbiol. 5:574. doi: 10.3389/fmicb.2014.00474, PMID: 25250022 PMC4158809

[ref6] Berleman J. E. Kirby J. R. (2007). Multicellular development in *Myxococcus xanthus* is stimulated by predator-prey interactions. J. Bacteriol. 189, 5675–5682. doi: 10.1128/JB.00544-07, PMID: 17513469 PMC1951827

[ref7] Berleman J. E. Scott J. Chumley T. Kirby J. R. (2008). Predataxis behavior in *Myxococcus xanthus*. Proc. Natl. Acad. Sci. U. S. A. 105, 17127–17132. doi: 10.1073/pnas.0804387105, PMID: 18952843 PMC2579389

[ref8] Chang Y. W. Rettberg L. A. Treuner-Lange A. Iwasa J. Søgaard-Andersen L. Jensen G. J. (2016). Architecture of the type IVa pilus machine. Science 351:aad2001. doi: 10.1126/science.aad2001, PMID: 26965631 PMC5929464

[ref9] Contreras-Moreno F. J. Muñoz-Dorado J. García-Tomsig N. I. Martínez-Navajas G. Pérez J. Moraleda-Muñoz A. (2020). Copper and melanin play a role in *Myxococcus xanthus* predation on *Sinorhizobium meliloti*. Front. Microbiol. 11:94. doi: 10.3389/fmicb.2020.00094, PMID: 32117124 PMC7010606

[ref10] Cortina N. S. Krug D. Plaza A. Revermann O. Müller R. (2012). Myxoprincomide: a natural product from *Myxococcus xanthus* discovered by comprehensive analysis of the secondary metabolome. Angew. Chem. Int. Ed. Engl. 51, 811–816. doi: 10.1002/anie.201106305, PMID: 22162209

[ref11] Costa T. R. Felisberto-Rodrigues C. Meir A. Prevost M. S. Redzej A. Trokter M.. (2015). Secretion systems in gram-negative bacteria: structural and mechanistic insights. Nat. Rev. Microbiol. 13, 343–359. doi: 10.1038/nrmicro3456 25978706

[ref12] Denise R. Abby S. S. Rocha E. P. C. (2019). Diversification of the type IV filament superfamily into machines for adhesion, protein secretion, DNA uptake, and motility. PLoS Biol. 17:e3000390. doi: 10.1371/journal.pbio.3000390, PMID: 31323028 PMC6668835

[ref13] Diepold A. Armitage J. P. (2015). Type III secretion systems: the bacterial flagellum and the injectisome. Philos. Philos. Trans. R. Soc. Lond. B Biol. Sci. 370, 20150020–20150119. doi: 10.1098/rstb.2015.0020, PMID: 26370933 PMC4632597

[ref14] Dong Y. Dong H. Feng Z. Wang X. Yao Q. Zhu H. (2022b). A disturbed siderophore transport inhibits myxobacterial predation. Cell 11:3718. doi: 10.3390/cells11233718, PMID: 36496980 PMC9738627

[ref15] Dong H. Gao R. Dong Y. Yao Q. Zhu H. (2023). Whole-genome sequencing of a biocontrol *Myxococcus xanthus* R31 isolate and comparative genomic analysis. Gene 863:147286. doi: 10.1016/j.gene.2023.147286, PMID: 36804855

[ref16] Dong H. Xu X. Gao R. Li Y. Li A. Yao Q.. (2022a). *Myxococcus xanthus* R31 suppresses tomato bacterial wilt by inhibiting the pathogen *Ralstonia solanacearum* with secreted proteins. Front. Microbiol. 12:801091. doi: 10.3389/fmicb.2021.801091, PMID: 35197943 PMC8859152

[ref17] Dongre M. Singh B. Aung K. M. Larsson P. Miftakhova R. Persson K.. (2018). Flagella-mediated secretion of a novel *Vibrio cholerae* cytotoxin affecting both vertebrate and invertebrate hosts. Commun. Biol. 1:59. doi: 10.1038/s42003-018-0065-z, PMID: 30271941 PMC6123715

[ref18] Ellison C. K. Kan J. Dillard R. S. Kysela D. T. Ducret A. Berne C.. (2017). Obstruction of pilus retraction stimulates bacterial surface sensing. Science 358, 535–538. doi: 10.1126/science.aan5706, PMID: 29074778 PMC5805138

[ref19] Evans A. G. Davey H. M. Cookson A. Currinn H. Cooke-Fox G. Stanczyk P. J.. (2012). Predatory activity of *Myxococcus xanthus* outer-membrane vesicles and properties of their hydrolase cargo. Microbiology 158, 2742–2752. doi: 10.1099/mic.0.060343-0, PMID: 22977088

[ref20] Galán J. E. Waksman G. (2018). Protein-injection machines in bacteria. Cell 172, 1306–1318. doi: 10.1016/j.cell.2018.01.034, PMID: 29522749 PMC5849082

[ref21] Goldman B. S. Nierman W. C. Kaiser D. Slater S. C. Durkin A. S. Eisen J. A.. (2006). Evolution of sensory complexity recorded in a myxobacterial genome. Proc. Natl. Acad. Sci. U. S. A. 103, 15200–15205. doi: 10.1073/pnas.0607335103, PMID: 17015832 PMC1622800

[ref22] Halte M. Erhardt M. (2021). Protein export via the type III secretion system of the bacterial flagellum. Biomol. Ther. 11, 186–219. doi: 10.3390/biom11020186, PMID: 33572887 PMC7911332

[ref23] Hart B. A. Zahler S. A. (1966). Lytic enzyme produced by *Myxococcus xanthus*. J. Bacteriol. 92, 1632–1637. doi: 10.1128/JB.92.6.1632-1637.1966, PMID: 5958103 PMC316242

[ref24] Hoffmann M. Auerbach D. Panter F. Hoffmann T. Dorrestein P. C. Müller R. (2018). Homospermidine lipids: a compound class specifically formed during fruiting body formation of *Myxococcus xanthus* DK1622. ACS Chem. Biol. 13, 273–280. doi: 10.1021/acschembio.7b00816, PMID: 29185703

[ref25] Islam S. T. Jolivet N. Y. Cuzin C. Belgrave A. M. My L. Fleuchot B.. (2023). Unmasking of the von Willebrand A-domain surface adhesin CglB at bacterial focal adhesions mediates myxobacterial gliding motility. Sci. Adv. 9:eabq0619. doi: 10.1126/sciadv.abq0619, PMID: 36812310 PMC9946355

[ref26] Kahnt J. Aguiluz K. Koch J. Treuner-Lange A. Konovalova A. Huntley S.. (2010). Profiling the outer membrane proteome during growth and development of the social bacterium *Myxococcus xanthus* by selective biotinylation and analyses of outer membrane vesicles. J. Proteome Res. 9, 5197–5208. doi: 10.1021/pr1004983, PMID: 20687614

[ref27] Konovalova A. Petters T. Søgaard-Andersen L. (2010). Extracellular biology of *Myxococcus xanthus*. FEMS Microbiol. Rev. 34, 89–106. doi: 10.1111/j.1574-6976.2009.00194.x, PMID: 19895646

[ref28] La Fortezza M. Rendueles O. Keller H. Velicer G. J. (2022). Hidden paths to endless forms most wonderful: ecology latently shapes evolution of multicellular development in predatory bacteria. Commun. Biol. 5:977. doi: 10.1038/s42003-022-03912-w, PMID: 36114258 PMC9481553

[ref29] Ladomersky E. Petris M. J. (2015). Copper tolerance and virulence in bacteria. Metallomics 7, 957–964. doi: 10.1039/c4mt00327f, PMID: 25652326 PMC4464932

[ref30] Lee N. Kim W. Chung J. Lee Y. Cho S. Jang K. S.. (2020). Iron competition triggers antibiotic biosynthesis in *Streptomyces coelicolor* during coculture with *Myxococcus xanthus*. ISME J. 14, 1111–1124. doi: 10.1038/s41396-020-0594-6, PMID: 31992858 PMC7174319

[ref31] Li Y. P. Ben Fekih I. Chi Fru E. Moraleda-Muñoz A. Li X. Rosen B. P.. (2021). Antimicrobial activity of metals and metalloids. Annu. Rev. Microbiol. 75, 175–197. doi: 10.1146/annurev-micro-032921-123231, PMID: 34343021 PMC8862609

[ref32] Livingstone P. G. Millard A. D. Swain M. T. Whitworth D. E. (2018). Transcriptional changes when *Myxococcus xanthus* preys on *Escherichia coli* suggest myxobacterial predators are constitutively toxic but regulate their feeding. Microb. Genom. 4:e000152. doi: 10.1099/mgen.0.000152, PMID: 29345219 PMC5857379

[ref33] Livingstone P. G. Morphew R. M. Whitworth D. E. (2017). Myxobacteria are able to prey broadly upon clinically-relevant pathogens, exhibiting a prey range which cannot be explained by phylogeny. Front. Microbiol. 8:1593. doi: 10.3389/fmicb.2017.01593, PMID: 28878752 PMC5572228

[ref34] Lloyd D. G. Whitworth D. E. (2017). The Myxobacterium *Myxococcus xanthus* can sense and respond to the quorum signals secreted by potential prey organisms. Front. Microbiol. 8:439. doi: 10.3389/fmicb.2017.00439, PMID: 28352265 PMC5348527

[ref35] McBride M. J. Zusman D. R. (1996). Behavioral analysis of single cells of *Myxococcus xanthus* in response to prey cells of *Escherichia coli*. FEMS Microbiol. Lett. 137, 227–231. doi: 10.1111/j.1574-6968.1996.tb08110.x, PMID: 8998990

[ref1001] Mendes-Soares H. Velicer G. J. (2013). Decomposing predation: testing for parameters that correlate with predatory performance by a social bacterium. Microb. Ecol. 65, 415–423. doi: 10.1007/s00248-012-0135-6 23184156 PMC3563865

[ref36] Moraleda-Muñoz A. Pérez J. Extremera A. L. Muñoz-Dorado J. (2010b). Differential regulation of six heavy metal efflux systems in the response of *Myxococcus xanthus* to copper. Appl. Environ. Microbiol. 76, 6069–6076. doi: 10.1128/AEM.00753-10, PMID: 20562277 PMC2937488

[ref37] Moraleda-Muñoz A. Pérez J. Extremera-León A. L. Muñoz-Dorado J. (2010a). Expression and physiological role of three *Myxococcus xanthus* copper-dependent P1B-type ATPases during bacterial growth and development. Appl. Environ. Microbiol. 76, 6077–6084. doi: 10.1128/AEM.00755-10, PMID: 20656859 PMC2937514

[ref38] Müller S. Strack S. N. Ryan S. E. Shawgo M. Walling A. Harris S.. (2016). Identification of functions affecting predator-prey interactions between *Myxococcus xanthus* and *Bacillus subtilis*. J. Bacteriol. 198, 3335–3344. doi: 10.1128/JB.00575-16, PMID: 27698086 PMC5116937

[ref39] Muñoz-Dorado J. Marcos-Torres F. J. García-Bravo E. Moraleda-Muñoz A. Pérez J. (2016). Myxobacteria: moving, killing, feeding, and surviving together. Front. Microbiol. 7:781. doi: 10.3389/fmicb.2016.00781, PMID: 27303375 PMC4880591

[ref40] Nair R. R. Vasse M. Wielgoss S. Sun L. Yu Y. N. Velicer G. J. (2019). Bacterial predator-prey coevolution accelerates genome evolution and selects on virulence-associated prey defences. Nat. Commun. 10:4301. doi: 10.1038/s41467-019-12140-6, PMID: 31541093 PMC6754418

[ref41] Pérez J. Contreras-Moreno F. J. Marcos-Torres F. J. Moraleda-Muñoz A. Muñoz-Dorado J. (2020). The antibiotic crisis: how bacterial predators can help. Comput. Struct. Biotechnol. J. 18, 2547–2555. doi: 10.1016/j.csbj.2020.09.010, PMID: 33033577 PMC7522538

[ref42] Pérez J. Contreras-Moreno F. J. Muñoz-Dorado J. Moraleda-Muñoz A. (2022). Development versus predation: transcriptomic changes during the lifecycle of *Myxococcus xanthus*. Front. Microbiol. 13:1004476. doi: 10.3389/fmicb.2022.1004476, PMID: 36225384 PMC9548883

[ref43] Pérez J. Jiménez-Zurdo J. I. Martínez-Abarca F. Millán V. Shimkets L. J. Muñoz-Dorado J. (2014). Rhizobial galactoglucan determines the predatory pattern of *Myxococcus xanthus* and protects *Sinorhizobium meliloti* from predation. Environ. Microbiol. 16, 2341–2350. doi: 10.1111/1462-2920.12477, PMID: 24707988 PMC4079745

[ref44] Pérez J. Moraleda-Muñoz A. Marcos-Torres F. J. Muñoz-Dorado J. (2016). Bacterial predation: 75 years and counting! Environ. Microbiol. 18, 766–779. doi: 10.1111/1462-2920.13171, PMID: 26663201

[ref45] Pérez J. Muñoz-Dorado J. Moraleda-Muñoz A. (2018). The complex global response to copper in the multicellular bacterium *Myxococcus xanthus*. Metallomics 10, 876–886. doi: 10.1039/c8mt00121a, PMID: 29961779

[ref46] Petters S. Groß V. Söllinger A. Pichler M. Reinhard A. Bengtsson M. M.. (2021). The soil microbial food web revisited: predatory myxobacteria as keystone taxa? ISME J. 15, 2665–2675. doi: 10.1038/s41396-021-00958-2, PMID: 33746204 PMC8397742

[ref47] Pham V. D. Shebelut C. W. Diodati M. E. Bull C. T. Singer M. (2005). Mutations affecting predation ability of the soil bacterium *Myxococcus xanthus*. Microbiology 151, 1865–1874. doi: 10.1099/mic.0.27824-0, PMID: 15941994

[ref48] Remis J. P. Wei D. Gorur A. Zemla M. Haraga J. Allen S.. (2014). Bacterial social networks: structure and composition of *Myxococcus xanthus* outer membrane vesicle chains. Environ. Microbiol. 16, 598–610. doi: 10.1111/1462-2920.12187, PMID: 23848955 PMC4234120

[ref49] Rensing C. McDevitt S. F. (2013). The copper metallome in prokaryotic cells. Met. Ions Life Sci. 12, 417–450. doi: 10.1007/978-94-007-5561-1_12, PMID: 23595679

[ref50] Rombouts S. Mas A. Le Gall A. Fiche J. B. Mignot T. Nollmann M. (2023). Multi-scale dynamic imaging reveals that cooperative motility behaviors promote efficient predation in bacteria. Nat. Commun. 14:5588. doi: 10.1038/s41467-023-41193-x, PMID: 37696789 PMC10495355

[ref51] Sánchez-Sutil M. C. Gómez-Santos N. Moraleda-Muñoz A. Martins L. O. Pérez J. Muñoz-Dorado J. (2007). Differential expression of the three multicopper oxidases from *Myxococcus xanthus*. J. Bacteriol. 189, 4887–4898. doi: 10.1128/JB.00309-07, PMID: 17483223 PMC1913447

[ref52] Seef S. Herrou J. de Boissier P. My L. Brasseur G. Robert D.. (2021). A tad-like apparatus is required for contact-dependent prey killing in predatory social bacteria. elife 10:e72409. doi: 10.7554/elife.72409 34505573 PMC8460266

[ref53] Skotnicka D. Søgaard-Andersen L. (2017). Type IV pili-dependent motility as a tool to determine the activity of c-di-GMP modulating enzymes in *Myxococcus xanthus*. Methods Mol. Biol. 1657, 157–165. doi: 10.1007/978-1-4939-7240-1_13, PMID: 28889293

[ref54] Soto M. J. Pérez J. Muñoz-Dorado J. Contreras-Moreno F. J. Moraleda-Muñoz A. (2023). Transcriptomic response of *Sinorhizobium meliloti* to the predatory attack of *Myxococcus xanthus*. Front. Microbiol. 14:1213659. doi: 10.3389/fmicb.2023.1213659, PMID: 37405170 PMC10315480

[ref55] Sudo S. Dworkin M. (1972). Bacteriolytic enzymes produced by *Myxococcus xanthus*. J. Bacteriol. 110, 236–245. doi: 10.1128/JB.110.1.236-245.1972, PMID: 4622898 PMC247403

[ref56] Sydney N. Swain M. T. So J. M. T. Hoiczyk E. Tucker N. P. Whitworth D. E. (2021). The genetics of prey susceptibility to Myxobacterial predation: a review, including an investigation into *Pseudomonas aeruginosa* mutations affecting predation by *Myxococcus xanthus*. Microb. Physiol. 31, 57–66. doi: 10.1159/000515546, PMID: 33794538

[ref57] Thiery S. Kaimer C. (2022). The predation strategy of *Myxococcus xanthus*. Front. Microbiol. 11:2. doi: 10.3389/fmicb.2020.00002, PMID: 32010119 PMC6971385

[ref58] Thiery S. Turowski P. Berleman J. E. Kaimer C. (2022). The predatory soil bacterium *Myxococcus xanthus* combines a tad -and an atypical type 3-like protein secretion system to kill bacterial cells. Cell Rep. 40:111340. doi: 10.1016/j.celrep.2022, PMID: 36103818

[ref59] Volz C. Kegler C. Müller R. (2012). Enhancer binding proteins act as hetero-oligomers and link secondary metabolite production to myxococcal development, motility, and predation. Chem. Biol. 19, 1447–1459. doi: 10.1016/j.chembiol.2012.09.010, PMID: 23177199

[ref60] Wagner S. Grin I. Malmsheimer S. Singh N. Torres-Vargas C. E. Westerhausen S. (2018). Bacterial type III secretion systems: a complex device for the delivery of bacterial effector proteins into eukaryotic host cells. FEMS Microbiol. Lett. 365, 1–13. doi: 10.1093/femsle/fny201, PMID: 30107569 PMC6140923

[ref61] Wall D. (2016). Kin recognition in bacteria. Annu. Rev. Microbiol. 70, 143–160. doi: 10.1146/annurev-micro-102215-095325, PMID: 27359217 PMC5759769

[ref62] Wang C. Xiao Y. Wang Y. Liu Y. Yao Q. Zhu H. (2023). Comparative genomics and transcriptomics insight into myxobacterial metabolism potentials and multiple predatory strategies. Front. Microbiol. 14:1146523. doi: 10.3389/fmicb.2023.1146523, PMID: 37213496 PMC10196010

[ref63] Whitworth D. E. Pérez J. Mitchell R. J. Pande S. (2023). Editorial: mechanisms of prokaryotic predation, volume II. Front. Microbiol. 14:1256252. doi: 10.3389/fmicb.2023.1256252, PMID: 37577415 PMC10415094

[ref64] Xiao Y. Gerth K. Müller R. Wall D. (2012). Myxobacterium-produced antibiotic TA (myxovirescin) inhibits type II signal peptidase. Antimicrob. Agents Chemother. 56, 2014–2021. doi: 10.1128/AAC.06148-11, PMID: 22232277 PMC3318312

[ref65] Xiao Y. Wei X. Ebright R. Wall D. (2011). Antibiotic production by myxobacteria plays a role in predation. J. Bacteriol. 193, 4626–4633. doi: 10.1128/jb.05052-11, PMID: 21764930 PMC3165673

[ref66] Zhang W. Wang Y. Lu H. Liu Q. Wang C. Hu W.. (2020). Dynamics of solitary predation by *Myxococcus xanthus* on *Escherichia coli* observed at the single-cell level. Appl. Environ. Microbiol. 86:e02286-19-13. doi: 10.1128/AEM.02286-19 31704687 PMC6974655

[ref67] Zwarycz A. S. Page T. Nikolova G. Radford E. J. Whitworth D. E. (2023). Predatory strategies of *Myxococcus xanthus*: prey susceptibility to OMVs and moonlighting enzymes. Microorganisms 11:874. doi: 10.3390/microorganisms11040874, PMID: 37110297 PMC10141889

